# Dynamic three-dimensional chromatin remodeling in lung disease: mechanistic principles, pathogenic regulatory networks, and translational challenges

**DOI:** 10.3389/fcell.2026.1943977

**Published:** 2026-09-30

**Authors:** Tiantian Liu, Songshan Jiang, Jing Wang, Jiansheng Li

**Affiliations:** 1 Henan Key Laboratory of Chinese Medicine for Respiratory Disease, Henan University of Chinese Medicine, Zhengzhou, Henan, China; 2 Co-construction Collaborative Innovation Center for Chinese Medicine and Respiratory Diseases by Henan & Education Ministry of P.R. China, Zhengzhou, Henan, China; 3 Academy of Chinese Medical Sciences, Henan University of Chinese Medicine, Zhengzhou, Henan, China

**Keywords:** 3D genome, chromatin remodeling, epigenetic regulation, lung disease, three-dimensional chromatin architecture

## Abstract

Three-dimensional (3D) chromatin architecture provides a spatial framework through which genomic sequences, epigenetic states, transcription factors, and distal regulatory elements are integrated to control cell type-specific gene expression. Increasing evidence suggests that disruption of this organization contributes to abnormal transcriptional programs in lung development and disease. In this review, we provide a mechanism-centered synthesis of 3D genome regulation in pulmonary biology, focusing on hierarchical features including A/B compartments, topologically associating domains, chromatin loops, and enhancer–promoter interactions. We discuss how architectural proteins, transcription factors, epigenetic modifications, and regulatory RNAs influence these structures and examine their involvement in chronic obstructive pulmonary disease, pulmonary fibrosis, lung cancer, and developmental lung abnormalities. Particular emphasis is placed on distinguishing direct evidence of altered chromatin architecture from changes in chromatin accessibility, transcription-factor activity, or epigenetic state that provide regulatory context but do not independently demonstrate 3D genome remodeling. Cross-disease comparison reveals both shared and context-dependent mechanisms, with altered long-range regulatory communication emerging as a recurrent feature while the relevant architectural scale, regulatory factors, cell types, and phenotypic consequences remain disease specific. We further discuss current limitations, including cellular heterogeneity, limited temporal resolution, and the challenge of establishing causality from static interaction maps. Advances in cell type-resolved chromatin conformation profiling, spatial multi-omics, live-cell imaging, and targeted perturbation should enable more precise mechanistic models and clarify whether disease-associated chromatin interactions can ultimately support patient stratification and therapeutic intervention in pulmonary disease.

## 1 Introduction

Three-dimensional (3D) chromatin architecture refers to the spatial folding, positioning, looping, and interaction patterns of chromatin within the nucleus. This architecture determines not only the territorial distribution of chromosomes but also the formation of multiple hierarchical functional units, including A/B compartments, topologically associating domains, and chromatin loops. These structural layers regulate gene expression by modulating the spatial proximity between genes and distal regulatory elements, such as enhancers and silencers, thereby enabling precise temporal and cell type-specific transcriptional control (Lieberman-Aiden et al., 2009; Rao et al., 2014; Rowley and Corces, 2018).

The 3D genome is highly dynamic rather than static. It is continuously remodeled during cell-cycle progression, lineage specification, organ development, and responses to environmental or pathological stimuli. High-throughput chromosome conformation capture technologies, particularly Hi-C and its derivatives, such as promoter-capture Hi-C, HiChIP, and single-cell Hi-C, have provided powerful tools for systematically investigating chromatin conformation changes and their roles in gene regulation (Yang et al., 2025a). Dynamic remodeling of spatial genome organization can coordinate the expression of gene clusters, thereby influencing cell fate decisions and the establishment of tissue-specific functions (Saw et al., 2025). An important conceptual distinction is required between 3D chromatin organization and dynamic 3D chromatin remodeling. Here, we use “3D chromatin architecture” to describe the spatial organization measured at a defined biological state or time point, whereas “3D chromatin remodeling” refers to changes in this architecture between biological states or over time. More stringently, *bona fide* dynamic remodeling requires evidence of temporal behavior obtained through longitudinal or time-course sampling, perturbation–recovery experiments, lineage-resolved analysis, or live-cell imaging (Dekker et al., 2017; Dekker et al., 2023). Many current studies of lung disease compare diseased and control tissues at a single endpoint and therefore demonstrate disease-associated or state-dependent 3D chromatin reorganization rather than directly resolving chromatin kinetics. This distinction is important when evaluating whether altered genome topology is a cause, consequence, or parallel correlate of transcriptional reprogramming.

In recent years, 3D genome biology has become an increasingly important area in respiratory disease research. Growing evidence suggests that the development and progression of lung diseases are accompanied not only by linear genomic alterations and conventional epigenetic changes but also by substantial reprogramming of chromatin spatial organization. Recent reviews have summarized Hi-C-based target discovery and potential therapeutic applications of three-dimensional genomics in pulmonary diseases (Yang B. et al., 2025). The present review is intended as a complementary, mechanism-centered synthesis. Rather than cataloguing disease-associated targets alone, we focus on how distinct levels of genome organization-including compartmentalization, TAD insulation, chromatin loops, and enhancer-promoter contacts-are dynamically remodeled; how architectural proteins, epigenetic states, and regulatory RNAs contribute to these processes; and how the strength of mechanistic inference differs between direct conformation measurements and functional-genomic evidence. We further emphasize unresolved causal direction, temporal dynamics, cellular heterogeneity, and functional perturbation as key requirements for translating spatial genome biology into mechanistically informed precision medicine.

In this review, we summarize the molecular basis of dynamic 3D chromatin remodeling and discuss its abnormal features and pathological functions in lung diseases. We focus particularly on its roles in inflammatory regulation, epithelial-mesenchymal transition, fibrotic remodeling, and tumor-associated transcriptional reprogramming. We also discuss current technical and conceptual challenges and future directions for translating spatial genome biology into precision medicine for lung diseases.

## 2 Molecular basis of dynamic 3D chromatin remodeling

The mammalian genome is organized in a multilayered spatial manner to support precise and coordinated gene regulation. The molecular basis of dynamic 3D chromatin remodeling includes chromatin compartment switching, formation and reorganization of topologically associating domains, establishment and rewiring of chromatin loops, and the coordinated actions of architectural proteins and epigenetic regulators.

### 2.1 Chromatin compartment switching and its regulatory mechanisms

Chromatin compartmentalization represents a major layer of 3D genome organization and broadly partitions chromatin into transcriptionally active A compartments and relatively inactive B compartments. Compartment identity correlates with gene density, transcriptional activity, chromatin accessibility, replication timing, histone modifications, and nuclear positioning (Lieberman-Aiden et al., 2009; Rao et al., 2014; Rowley and Corces, 2018). Current evidence indicates that A/B compartments are not generated primarily by the CTCF-cohesin loop-extrusion machinery. Instead, compartmentalization is thought to arise from preferential interactions among chromatin regions with similar biochemical states, together with their differential association with nuclear environments such as the nuclear lamina and transcription-associated nuclear bodies (Rowley and Corces, 2018; Falk et al., 2019). Experimental and polymer-modeling studies further support an important contribution of heterochromatin self-affinity to the segregation of active and inactive chromatin (Falk et al., 2019).

This mechanism is distinct from CTCF/cohesin-dependent local insulation. Acute CTCF depletion strongly disrupts CTCF-anchored loops and TAD insulation while leaving higher-order A/B compartment segregation largely intact (Nora et al., 2017). Similarly, acute cohesin depletion eliminates loop domains but does not abolish compartmentalization and may increase segregation between chromatin states (Rao et al., 2017; Schwarzer et al., 2017). These findings support a model in which chromatin-state-dependent compartment formation and cohesin-mediated loop extrusion constitute partially separable organizing principles of the mammalian genome.

Dynamic A-to-B or B-to-A compartment switching is frequently accompanied by changes in transcription, histone modifications, and accessibility. Allele-specific 3D genome analyses further show that differences in chromatin organization between homologous alleles can correlate with allele-specific epigenetic states. The relevance of this phenomenon to lung disease lies in its ability to connect cis-acting genetic variation with regulatory chromatin state and spatial gene control. Because many lung-disease-associated variants are located in non-coding regions, an allele may alter transcription-factor occupancy or enhancer activity and thereby influence long-range regulation of a disease-relevant target gene. Functional studies at the chronic obstructive pulmonary disease (COPD) -associated HHIP locus and other non-coding lung-disease loci illustrate this general principle (Stuart et al., 2020; Guo et al., 2024). Nevertheless, direct allele-resolved 3D genome maps from primary human lung disease tissues remain limited. Allele-specific architecture should therefore be regarded as an important emerging mechanism and research opportunity rather than an established universal feature of lung disease.

### 2.2 Structural features and dynamic regulation of TADs

Topologically associating domains (TADs) are genomic regions in which chromatin contacts occur more frequently within the domain than across neighboring domains. In mammalian cells, many prominent loop domains and insulated TAD boundaries are strongly influenced by cohesin-mediated loop extrusion and convergently oriented CTCF-binding sites (Rao et al., 2014; Nora et al., 2017; Rao et al., 2017; Schwarzer et al., 2017). However, TAD-like domains should not be considered products of a single mechanism. Domains associated with compartmental segregation or chromatin-state transitions can persist even when CTCF or cohesin is acutely depleted, indicating that local insulation and biochemical compartmentalization represent partially independent layers of genome organization (Nora et al., 2017; Rao et al., 2017; Schwarzer et al., 2017; Rowley and Corces, 2018).

Studies have shown that TADs are typically formed by chromatin loops anchored by CTCF and the cohesin complex (Hansen et al., 2019). The integrity of TAD boundaries is essential for maintaining a stable local gene regulatory environment, and this stability is highly dependent on CTCF. In the erythroid lineage, loss of CTCF leads to disruption of TAD boundaries and collapse of chromatin loops (Yang et al., 2025b). In addition, CTCF motifs are directionally oriented, a property that determines the termination sites of chromatin loop extrusion and thereby helps maintain loop stability. Notably, TADs often contain multiple sub-TADs, whose boundaries are also enriched for CTCF-binding sites. Deletion of boundary-associated CTCF motifs disrupts local domain architecture and can reduce transcriptional activity of genes within the domain (Lee et al., 2024). CTCF-dependent boundaries restrict inappropriate communication between regulatory elements located in neighboring genomic domains, thereby contributing to enhancer–promoter specificity. Experimental deletion of selected boundary-associated CTCF sites can weaken local insulation, merge regulatory environments, or alter transcription of nearby genes. Importantly, however, not every change in a TAD boundary produces a transcriptional phenotype, and the functional consequence depends on the regulatory elements and genes located on either side of the affected boundary. Thus, TAD architecture should be interpreted as a context-dependent constraint on regulatory communication rather than as an invariant determinant of transcription.

### 2.3 Chromatin loop formation and enhancer–promoter interactions

CTCF is a key architectural protein enriched at TAD boundaries and involved in chromatin loop formation. Genome-wide chromatin interaction analyses have shown that CTCF–CTCF interactions constitute a major structural basis of chromatin loops (Galan et al., 2022). TADs and chromatin loops substantially influence the efficiency and specificity of enhancer–promoter interactions, thereby determining transcriptional activity and regulatory specificity. During embryonic stem cell differentiation, TAD boundaries and chromatin loop structures are progressively reinforced, indicating that topological remodeling plays an important role in developmental gene regulation (Dehingia et al., 2025). Experimental studies using different transposon insertion systems have further revealed scale-dependent features of chromatin looping: murine leukemia virus preferentially integrates into smaller enhancer–promoter loops, whereas piggyBac transposons may span larger loops across entire TADs (Yoshida et al., 2017). In addition, SATB1 and CTCF broadly co-localize across the genome. Loss of SATB1 enhances chromatin contacts between shared binding sites and affects the transcription of key regulatory factors in T cells (Wang et al., 2023). These findings indicate that chromatin loops are not merely structural units but also functional platforms for gene regulatory network formation.

### 2.4 Key architectural regulators maintaining 3D genome structure

CTCF and cohesin are central components of the loop-extrusion system that organizes many mammalians chromatin loops and insulated domains. CTCF is a sequence-specific zinc-finger DNA-binding protein that occupies numerous loop anchors and TAD boundaries. The orientation of CTCF motifs is important because many cohesin-dependent loops preferentially form between convergently oriented CTCF sites (Rao et al., 2014). Acute CTCF depletion causes extensive loss of local insulation and CTCF-anchored loops, whereas higher-order compartmental segregation is comparatively preserved (Nora et al., 2017). These findings indicate that CTCF primarily contributes to the positioning and stabilization of extrusion-dependent boundaries rather than serving as a universal determinant of all levels of chromatin organization.

Cohesin is an ATPase-containing structural maintenance of chromosomes complex whose core components include SMC1/SMC3, RAD21, and STAG1 or STAG2. The NIPBL-MAU2 complex promotes cohesin loading and stimulates loop extrusion, whereas WAPL/PDS5-associated mechanisms contribute to cohesin release and residence-time control (Davidson et al., 2019; Davidson and Peters, 2021). Biochemical reconstitution has demonstrated that human cohesin together with NIPBL–MAU2 can actively extrude DNA loops in an ATPase-dependent manner (Davidson et al., 2019). In this framework, cohesin functions as the principal extrusion machinery, whereas CTCF acts as a directional boundary that limits or stabilizes extrusion at selected genomic positions.

The dynamic nature of this system is particularly evident in acute cohesin depletion–recovery experiments. Loss of cohesin eliminates loop domains, whereas restoration of cohesin leads to rapid re-formation of many chromatin loops on a minute-to-hour timescale (Rao et al., 2017). These experiments provide a direct demonstration of temporal architectural remodeling and should be distinguished from cross-sectional comparisons that identify different chromatin states without measuring their kinetics. Alterations in CTCF, cohesin, STAG proteins, or their regulatory partners may therefore reshape local regulatory topology; however, because these proteins have broad and essential genomic functions, their pathological effects are likely to depend strongly on cellular context and locus-specific regulatory environments.

Taken together, current evidence supports a model in which mammalian 3D chromatin architecture emerges from at least two partially separable organizing processes: chromatin-state-dependent spatial segregation, which contributes strongly to A/B compartmentalization, and cohesin-mediated loop extrusion constrained by CTCF, which contributes to local loops and TAD insulation (Nora et al., 2017; Rao et al., 2017; Schwarzer et al., 2017; Davidson et al., 2019; Falk et al., 2019; Davidson and Peters, 2021). These processes interact with transcription factors, histone modifications, DNA methylation, chromatin accessibility, and nuclear positioning rather than operating independently. Consequently, disease-associated 3D genome reorganization can arise from altered chromatin state, altered architectural constraint, or both. Equally importantly, altered spatial contacts should not automatically be interpreted as causal. In some settings, topology constrains which enhancers can access a promoter; in others, transcription-factor activation and epigenetic remodeling may themselves modify contact probabilities. Distinguishing these possibilities requires perturbational and temporal experiments rather than contact maps alone.

### 2.5 Enhancer RNAs as regulators of 3D enhancer–promoter communication

Enhancer RNAs (eRNAs) are non-coding transcripts generated from transcriptionally active enhancers and represent an additional regulatory layer linking enhancer activity to gene expression (Sartorelli and Lauberth, 2020). Genome-wide analyses have shown that enhancer transcription is highly cell- and tissue-specific and is strongly associated with enhancer activation (Andersson et al., 2014). At selected loci, eRNAs can influence three-dimensional enhancer–promoter communication through interactions with transcriptional coactivators and architectural complexes. For example, activating non-coding RNAs were shown to interact with Mediator, and depletion of either the RNA or Mediator components reduced chromatin looping between regulatory loci and their target genes (Lai et al., 2013). Similarly, androgen receptor-induced eRNAs have been implicated in strengthening enhancer-promoter looping and target-gene activation (Hsieh et al., 2014). These effects are nevertheless context-dependent rather than universal. In neuronal immediate-early gene regulation, eRNA depletion impaired release of paused RNA polymerase II without disrupting the enhancer–promoter interaction, suggesting that pre-existing spatial proximity can also provide a framework within which eRNAs regulate transcription downstream of loop formation (Schaukowitch et al., 2014). Thus, eRNAs should be considered a context-dependent regulatory interface among enhancer activation, transcriptional coactivators, chromatin topology, and target-gene transcription.

These organizing principles provide a framework for interpreting pulmonary 3D-genome studies, but their disease relevance must be established directly in lung-relevant cells and tissues. Accordingly, the following sections prioritize studies in which compartments, TADs, loops, or enhancer–promoter contacts were measured in lung development or disease. Chromatin-accessibility, transcription-factor-binding, and other epigenomic datasets are used primarily to interpret the regulatory state of interacting regions rather than as evidence of 3D remodeling by themselves.

## 3 Aberrant 3D chromatin architecture in lung diseases

Epigenetic dysregulation is closely associated with multiple pulmonary diseases, and abnormal spatial genome organization represents an important component of this process. Alterations in 3D chromatin architecture can influence gene regulatory networks and thereby contribute to lung disease initiation and progression (Wang D. C. et al., 2019). Chromatin structural abnormalities may also affect genome stability, particularly in aging and disease contexts (Eremenko et al., 2021). In major lethal lung diseases, including COPD, pulmonary fibrosis, and lung cancer, aberrant 3D chromatin organization has increasingly been recognized as a critical pathological feature (Avci et al., 2022).

The strength and type of evidence are not evenly distributed among lung diseases. Lung cancer currently accounts for a substantial proportion of genome-wide and mechanistically interrogated studies of 3D chromatin regulation, whereas evidence in COPD, pulmonary fibrosis, and developmental lung disorders remains comparatively limited and often derives from selected loci, bulk tissues, or complementary epigenomic datasets. This imbalance is biologically and methodologically important. In the following sections, we therefore distinguish direct evidence of 3D chromatin organization, obtained by chromosome-conformation or spatial imaging methods, from supporting regulatory evidence, such as chromatin accessibility, transcription-factor occupancy, or transcriptomic changes. The latter can identify candidate regulatory mechanisms but should not by itself be interpreted as proof of altered physical chromatin contacts.

### 3.1 3D chromatin alterations in chronic obstructive pulmonary disease

The onset and progression of COPD are closely associated with environmental exposures, genetic susceptibility, and epigenetic reprogramming. In this context, abnormalities in 3D chromatin architecture may represent a critical link between external stimuli and aberrant transcriptional responses (Yang et al., 2025a). Environmentally induced epigenetic responses can reshape cellular phenotypes and gene expression programs by altering spatial chromatin conformation.

Studies have shown that a distal enhancer within the chromosome 4q31 region can physically interact with the HHIP gene in a SMAD3-dependent manner, thereby regulating its transcription. Downregulation of HHIP enhances transforming growth factor-β (TGF-β)-induced epithelial–mesenchymal transition (EMT), suggesting a potential role in COPD pathogenesis (Guo et al., 2024). Functional interrogation of COPD risk loci using Hi-C, CRISPR interference (CRISPRi), and expression quantitative trait locus (eQTL) analyses further revealed that several disease-associated single-nucleotide polymorphisms, including SNP rs1800469 and rs35705950, can interact with nearby genes and function as enhancer elements to regulate the expression of *TGFB*, *MUC5AC*, and *MUC5B* (Stuart et al., 2020). These studies collectively support a mechanistic model in which non-coding COPD risk regions can influence disease-relevant transcription through cell type-specific distal regulatory interactions rather than through alterations in protein-coding sequence. The HHIP locus provides particularly informative evidence because deletion of the distal regulatory element not only reduces HHIP expression but also markedly perturbs local chromatin interactions, strengthening the inference that the enhancer contributes to both regulatory activity and local topology (Guo et al., 2024). At other lung-disease-associated loci, integration of Hi-C, eQTL, and epigenomic information can nominate candidate enhancer–gene relationships, whereas CRISPRi provides an essential functional step for distinguishing regulatory variants from variants that are merely located within correlated chromatin states (Stuart et al., 2020). For precision, rs1800469 should be linked to regulation of TGFB1, whereas rs35705950 has regulatory effects involving MUC5AC/MUC5B in the cited functional study (Stuart et al., 2020).

Nevertheless, the current COPD literature remains concentrated on a relatively small number of risk loci and epithelial models. It is not yet known whether chronic cigarette-smoke exposure, inflammatory stress, or repeated exacerbations induce reproducible and reversible genome-wide compartment or loop remodeling within individual primary lung cell types. Addressing this question will require longitudinal perturbation models and cell type-resolved 3D genome measurements rather than disease-control comparisons alone.

### 3.2 3D genome remodeling in pulmonary fibrosis

Pulmonary fibrosis is a progressive disease characterized by fibroblast activation, excessive extracellular matrix deposition, and destruction of normal lung architecture. Its pathological progression is closely associated with 3D genome reorganization. In idiopathic pulmonary fibrosis, abnormal spatial chromatin conformation may promote aberrant activation of profibrotic genes by altering enhancer–promoter interactions (Avci et al., 2022). In addition, nuclear envelope damage and disruption of the nuclear lamina can further perturb chromatin function at the molecular level and exacerbate genomic instability, potentially contributing together to the progression of pulmonary fibrosis (Karoutas and Akhtar, 2021). In a bleomycin-induced model of pulmonary fibrosis, integrative multi-omics analyses combining chromatin architecture, chromatin accessibility, and gene expression profiles systematically revealed key features of 3D genome remodeling during fibrotic progression. Recent integrative analysis of a bleomycin-induced pulmonary fibrosis model combined Hi-C, ATAC-seq, bulk RNA-seq, and single-cell RNA-seq and identified disease-associated differences in chromatin interaction patterns, A/B compartment status, accessibility, and gene expression (Li et al., 2025). The concordance of these layers supports a close relationship between chromatin state and fibrotic transcriptional programs, particularly those associated with inflammation and extracellular matrix remodeling. However, the Hi-C measurements were obtained from whole lung tissue at an experimental endpoint. They therefore demonstrate fibrosis-associated 3D chromatin reorganization but do not by themselves resolve the temporal kinetics of architectural remodeling during injury, inflammation, fibroblast activation, and resolution or progression.

This distinction is particularly important because fibrotic lungs undergo profound changes in cellular composition. A bulk increase or decrease in a chromatin contact may reflect a genuine architectural change within a cell type, an expansion of a cell population with a pre-existing chromatin configuration, or a combination of both. Recent single-nucleus ATAC/RNA multi-omics analysis of human systemic sclerosis-associated interstitial lung disease identified cell type-specific regulatory programs, including increased AP-1, RUNX, and EGR-associated enhancer activity in profibrotic fibroblasts and distinct AP-1/bHLH-ZIP-associated regulatory activity in SPP1-high macrophages (Valenzi et al., 2026). These observations provide important information about cell-specific regulatory states, but chromatin accessibility and inferred transcription-factor activity do not directly establish physical enhancer–promoter contacts. Future integration of such single-cell regulatory maps with cell type-resolved chromatin-conformation approaches will therefore be required to determine which profibrotic regulatory elements are linked through disease-specific 3D interactions.

Accordingly, current fibrosis data suggest that spatial genome reorganization is associated with fibrotic cell-state transitions, but several causal questions remain unresolved: whether architectural changes precede activation of profibrotic genes, whether they are maintained after removal of fibrotic stimuli, and whether the same changes occur in fibroblasts, epithelial cells, macrophages, and other disease-relevant populations.

### 3.3 3D chromatin dysregulation in lung cancer

In recent years, lung cancer research has expanded from a primary focus on linear genomic abnormalities to the spatial genome dimension. Accumulating evidence indicates that 3D chromatin architecture reprogramming is not merely a byproduct of lung cancer progression, but a key mechanism linking genomic alterations to aberrant transcriptional programs (Jing et al., 2026).

Dysregulated chromatin interactions are a major manifestation of 3D genome abnormalities in lung cancer. Studies have shown that regions surrounding lung cancer–related genes are embedded in complex chromatin interaction networks coordinated by multiple regulatory factors. When these interaction patterns become disrupted, distal enhancers may aberrantly activate inappropriate target genes or fail to support normal regulatory programs, ultimately leading to oncogenic imbalance in gene expression (Zhang Y. et al., 2023). Integrative analyses of clinical lung cancer samples—combining copy number variation, point mutation, and three-dimensional3D genome profiling—have shown that somatic mutations, copy number abnormalities, and structural variants affect not only the DNA sequence itself, but also chromatin compartment status, local topological organization, and spatial contact patterns. These alterations can establish novel oncogenic regulatory circuits, thereby promoting oncogene activation and/or tumor suppressor gene silencing (Li et al., 2021). 3D genome reprogramming in lung cancer is locus-specific and dynamically plastic. Together with local chromatin states, it cooperatively shapes transcriptional regulation and may influence responses to epigenetic therapies (Venu et al., 2025).

In studies of treatment response, chromatin accessibility in lung cancer cells has been shown to change dynamically following chemotherapy. ATAC-seq analyses revealed that genomic regions differentially accessible between chemotherapy-induced dormant cells and reactivated cells are significantly enriched for enhancer elements and binding motifs associated with key transcription factors, including JUN, MYC, SMAD3, E2F1, SP1, CTCF, SMAD4, STAT3, NFKB1, and KLF4. These factors likely contribute to transcriptional reprogramming during dormancy and reactivation by modulating promoter accessibility of their target genes (Wang et al., 2021). In addition, overexpression of the lncRNA IGF1R-AS1 promotes long-range chromatin looping between a distal MYC enhancer and the MYC promoter through interactions with chromatin-remodeling complexes and structural proteins, thereby driving MYC overexpression and enhancing tumorigenic capacity (Yang et al., 2026). The glucocorticoid receptor (GR) exerts tumor-suppressive effects, and its coregulatory factors PAXIP1 and the cohesin subunit STAG2 are essential for maintaining 3D genome architecture and hormone-induced transcriptional programs. Notably, loss of PAXIP1/STAG2 reshapes local chromatin interactions and can potentiate GR-mediated tumor suppression (Mayayo-Peralta et al., 2023). Another study demonstrated that SMAD4 restricts chromatin loop formation by insulating EP300 from loop-anchor contacts. Loss of SMAD4 leads to increased H3K27ac deposition, aberrant SOX2 activation, and enhanced proliferation of lung squamous cell carcinoma cells (Tang et al., 2026b).

Recent studies provide substantially stronger direct evidence for multiscale 3D genome remodeling during lung tumor progression. Using single-cell spatial genome profiling in Kras-driven cancer models, Liu et al. traced stage-dependent alterations in chromatin compaction and A/B compartment organization during progression toward lung adenocarcinoma. RNF2 occupancy was enriched in regions undergoing progression-associated compartment changes, and both RNF2 knockdown and rapid dTAG-mediated RNF2 degradation produced corresponding changes in single-cell A/B compartment scores. These perturbational results support a direct role for RNF2 in at least part of the 3D genome reorganization accompanying lung adenocarcinoma progression (Liu et al., 2025a).

In a complementary model of radiation-induced malignant transformation of human bronchial epithelial cells, integrated Hi-C, Micro-C, ATAC-seq, RNA-seq, and CUT&Tag revealed B-to-A compartment switching, altered TAD organization, and tumor-specific chromatin loops. TP63 promoted formation of new accessible chromatin sites associated with increased CTCF and cohesin occupancy. At the MYC locus, a newly established TP63-associated CTCF site formed a novel TAD boundary and chromatin loop contacting the MYC promoter, accompanied by increased MYC expression. These observations directly connect transcription-factor-dependent chromatin opening with architectural-protein recruitment and tumor-specific 3D genome remodeling (Liu et al., 2025b).

Taken together, lung cancer provides the most extensive current evidence that oncogenic transcriptional reprogramming can be coupled to alterations in regulatory topology. However, the studies summarized above also illustrate that not all epigenomic changes represent equivalent evidence of 3D remodeling. For example, enrichment of JUN, MYC, SMAD3, STAT3, NFKB1, or CTCF motifs in differentially accessible regions identifies candidate regulators of treatment-associated transcriptional states (Wang et al., 2021), whereas direct demonstration of an altered chromatin loop, as reported for IGF1R-AS1-dependent MYC regulation or SMAD4-associated loop insulation, provides stronger structural evidence (Tang et al., 2026b; Yang et al., 2026). These levels of evidence should therefore be interpreted separately. A major future challenge is to determine which tumor-specific loops are causal and therapeutically required, which are consequences of oncogenic transcription, and which merely reflect changes in cell state or clonal composition.

### 3.4 Abnormal higher-order chromatin architecture in developmental lung disorders

Precisely organized 3D chromatin architecture is a fundamental basis for establishing refined transcriptional programs in complex organisms. Increasing evidence supports its essential role in development and suggests that its disruption may represent a major pathogenic mechanism in developmental diseases (Anania and Lupiáñez, 2020). During lung development, higher-order chromatin structure regulates the spatiotemporal expression of key gene clusters, thereby shaping lung organogenesis. Lung development proceeds through proximal–distal patterning and branching morphogenesis to generate a complex tree-like architecture. Single-cell multi-omics atlases have mapped lineage-determining transcription factors and gene regulatory networks that drive cell differentiation, demonstrating that the transcriptional regulator CTCF coordinates maintenance of lung progenitor cells and branching morphogenesis by modulating gene expression and chromatin accessibility (Sun et al., 2025). When chromatin compartment switching becomes aberrant or topologically associating domain (TAD) boundaries are disrupted, chromatin interaction patterns and gene expression programs are altered, which can lead to reduced embryonic viability and pronounced anatomical phenotypes. Notably, deletion of the TAD boundary near Smad3/Smad6 causes complete embryonic lethality, whereas deletion of the boundary near Tbx5/Lhx5 results in severe lung malformations (Rajderkar et al., 2023) (Figure 1).

**FIGURE 1:**
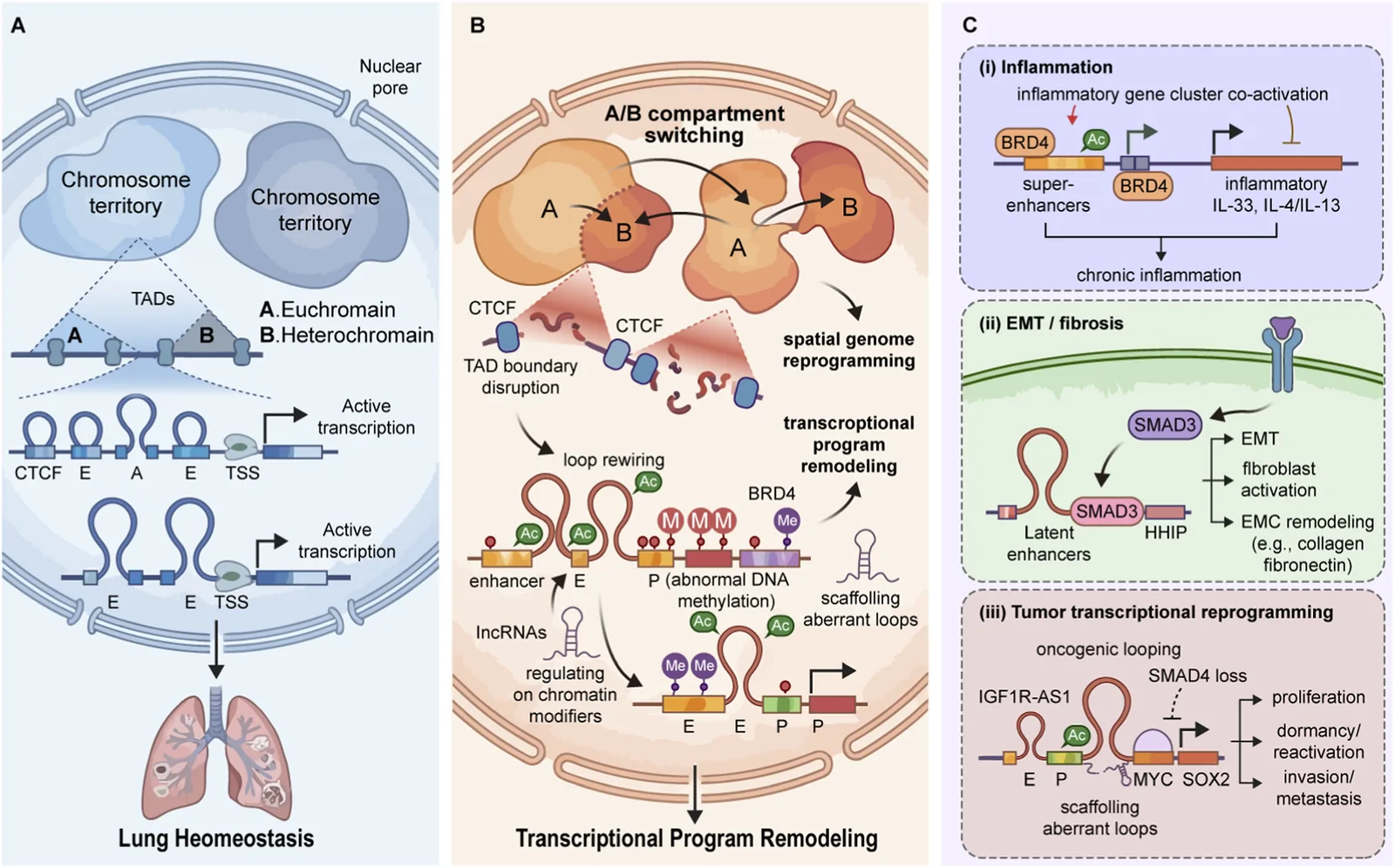
Dynamic remodeling of 3D chromatin architecture in the pathogenesis and progression of lung diseases. **(A)** Normal 3D genome organization. 3D chromatin architecture is organized into multiple hierarchical layers, including chromosome territories, A/B compartments, topologically associating domains, and chromatin loops. These structures maintain precise enhancer–promoter interactions, coordinate spatiotemporal gene expression, and preserve lung tissue homeostasis. Architectural proteins such as CTCF and cohesin stabilize TAD boundaries and anchor chromatin loops. **(B)** Dynamic chromatin remodeling. Environmental stimuli, genetic susceptibility, and epigenetic abnormalities can induce dynamic remodeling of 3D chromatin architecture, including A/B compartment switching, TAD boundary disruption, chromatin loop rewiring, and aberrant enhancer–promoter interactions. These changes are accompanied by altered DNA methylation, abnormal histone modifications, chromatin accessibility remodeling, and long non-coding RNA-mediated regulation, ultimately resulting in disease-associated transcriptional reprogramming. **(C)** Pathological regulatory networks. Persistent or aberrant remodeling of spatial regulatory interactions can contribute to disease-associated transcriptional programs involving inflammatory responses, epithelial-mesenchymal transition, fibrotic remodeling, and tumor-associated transcriptional reprogramming. The arrows indicate proposed regulatory relationships rather than a universal causal sequence; the relative contribution of each architectural level varies among cell types and disease contexts. CTCF: CCCTC-binding factor; Cohesin: Cohesin complex; TADs: Topologically associated domains; EMT: Epithelial-mesenchymal transition; COPD: Chronic obstructive pulmonary disease.

Single-cell epigenomic studies provide an important regulatory context for interpreting 3D genome organization during lung development, although most do not directly measure chromatin conformation. A human fetal lung atlas integrating scRNA-seq, scATAC-seq, spatial transcriptomics, and imaging identified cell-state-specific regulatory landscapes across epithelial, mesenchymal, endothelial, and immune compartments and experimentally validated transcription-factor programs controlling developmental cell fates (He et al., 2022). During postnatal alveologenesis, single-cell chromatin-accessibility profiling similarly identified regulatory modules associated with AT1 differentiation, myofibroblast states, and lung-function-associated genetic variants (Duong et al., 2022). In mouse alveolar epithelial development, NKX2-1 displays cell-state-specific chromatin binding, with YAP/TAZ contributing to AT1-specific NKX2-1 occupancy and cell-fate specification (Little et al., 2021). PRDM3 and PRDM16 further regulate chromatin accessibility at NKX2-1-associated regulatory elements required for alveolar epithelial differentiation and function (He et al., 2024). More recently, conditional deletion of Ctcf in mouse lung epithelium disrupted lineage-specific chromatin accessibility, progenitor maintenance, and branching morphogenesis (Sun et al., 2025). These studies demonstrate that cell-type-specific accessibility and architectural-protein occupancy are functionally important during lung development, but-with the exception of studies incorporating direct conformation measurements-they should not be interpreted as evidence for specific changes in TADs or chromatin loops.

Developmental studies provide an especially informative framework for evaluating causality because chromatin architecture can be perturbed *in vivo* before the resulting anatomical phenotype is assessed. Targeted deletion of selected TAD boundaries produces a spectrum of outcomes rather than a uniform phenotype; notably, deletion of the boundary near Smad3/Smad6 causes embryonic lethality, whereas deletion near Tbx5/Lhx5 produces severe lung abnormalities (Rajderkar et al., 2023). These findings demonstrate that selected boundaries can be functionally essential, but they also caution against assuming that every TAD boundary has equivalent regulatory importance. The biological consequence depends on developmental stage, cell lineage, and the enhancer-gene relationships constrained by the particular boundary. Future studies combining stage-resolved single-cell chromatin profiling with genetic boundary perturbation will be needed to identify which topological structures are instructive for lung morphogenesis and which primarily reflect lineage state.

## 4 Dynamic 3D chromatin remodeling in pathogenic regulatory networks of lung disease

Dynamic 3D chromatin remodeling should be understood as a regulatory process that links upstream disease stimuli to context-dependent transcriptional responses rather than as an isolated structural abnormality. Across COPD, pulmonary fibrosis, lung cancer, and developmental lung disorders, current evidence can be integrated into a common conceptual sequence: genetic or environmental perturbation, altered transcription-factor occupancy and epigenetic state, reorganization of spatial regulatory constraints, altered enhancer-promoter communication, disease-associated gene-expression programs, pathological phenotype. The relative contribution of each step, however, differs substantially among diseases and remains incompletely resolved.

At the biochemical level, disease-responsive transcription factors and chromatin architectural proteins perform complementary rather than interchangeable functions. Factors such as SMAD3/SMAD4, AP-1, NF-κB, STAT3, and MYC can alter enhancer activity, transcriptional competence, and chromatin accessibility, whereas CTCF and cohesin constrain the topology within which many distal regulatory interactions occur. Consequently, pathological transcription can emerge either through altered activity of regulatory elements within an otherwise similar topological domain or through rewiring of the domain itself. This distinction helps reconcile apparently different observations across lung diseases.

In COPD, current mechanistic evidence is particularly consistent with non-coding enhancer dysfunction within genetically susceptible loci, exemplified by SMAD3-dependent regulation of HHIP (Guo et al., 2024). In pulmonary fibrosis, available genome-wide data emphasize compartment switching, accessibility changes, and cell-state-specific transcription-factor programs associated with inflammatory and extracellular-matrix remodeling (Li et al., 2025; Valenzi et al., 2026). In lung cancer, structural variants, oncogenic signaling, lncRNAs, and altered architectural cofactors can create or stabilize ectopic enhancer–promoter interactions, providing a more direct route to oncogene activation (Li et al., 2021; Mayayo-Peralta et al., 2023; Tang et al., 2026a; Yang et al., 2026). During lung development, CTCF-dependent regulatory organization and selected TAD boundaries constrain developmental gene programs, with boundary disruption capable of producing severe *in vivo* phenotypes (Rajderkar et al., 2023; Sun et al., 2025). Thus, the same organizational principles are reused in different pathological contexts, but the affected genomic loci, regulatory factors, and phenotypic consequences remain disease- and cell type-specific.

### 4.1 Long-range chromatin interactions in inflammation-associated gene regulation

Inflammatory gene regulation is frequently described as a coordinated transcriptional process; however, direct evidence that multiple inflammation-related genes form common spatial clusters in lung disease remains limited. Current chromatin-conformation studies more strongly support locus-specific long-range regulatory interactions. At the asthma-associated 17q21 locus, the risk variants rs4065275 and rs12936231 alter CTCF binding, and 4C-seq demonstrated risk-allele-dependent contacts between distal cis-regulatory elements upstream of ZPBP2 and the ORMDL3 promoter in CD4^+^ T cells. ORMDL3 expression was in turn linked to altered IL-2 production, providing an example in which genotype-dependent chromatin architecture is associated with an inflammatory transcriptional phenotype (Schmiedel et al., 2016). In another study of chronic lung disease-associated loci, integration of Hi-C and epigenomic information with CRISPR interference demonstrated that the non-coding region containing rs1800469 functions as a regulatory element controlling TGFB1 expression in lung epithelial cells and primary lung fibroblasts (Stuart et al., 2020). These observations support a model in which disease-associated variants and architectural constraints influence specific long-range regulatory contacts that modulate inflammatory or remodeling-associated genes. They do not yet establish a general mechanism involving the spatial co-localization of multiple inflammation-related gene clusters.

### 4.2 3D genome reconfiguration during epithelial-mesenchymal transition

EMT is a key biological process underlying lung cancer invasion and metastasis, fibrotic tissue remodeling in the lung, and pathological airway remodeling. Among these contexts, EMT has been most extensively investigated in non-small cell lung cancer (NSCLC), where it endows tumor cells with enhanced migratory and invasive capacities, increased drug resistance, and stemness-associated traits, thereby directly promoting metastasis and recurrence (Dela Cruz and Medina, 2025). Direct chromatin-conformation evidence indicates that EMT-associated transcriptional reprogramming does not necessarily require extensive global reorganization of the 3D genome. In A549 cells undergoing TGF-β-induced EMT, Hi-C analysis showed that enhancer gain and loss occurred predominantly within pre-existing TADs, whereas genome-wide chromatin interaction patterns and higher-order topology were comparatively stable. Nevertheless, gained enhancers showed preferential interactions with TGF-β-upregulated genes, whereas lost enhancers were preferentially associated with downregulated genes, indicating that regulatory reprogramming can occur within an existing topological framework (Qiao et al., 2020). Consistent with such a locus-specific mechanism, a TGF-β-responsive enhancer located approximately 46 kb downstream of SNAI1 was shown to interact with the SNAI1 proximal promoter in A549 cells. CRISPRi-mediated inhibition of this enhancer attenuated TGF-β-induced SNAI1 expression, stress-fiber formation, and increased cell motility (Fu et al., 2025). Thus, the available evidence supports local enhancer reprogramming and enhancer–promoter regulation during EMT rather than a universal model of large-scale 3D genome reconfiguration.

### 4.3 Coordinated regulation between 3D genome architecture and epigenetic regulation

Chromatin accessibility, histone modifications, and transcription-factor occupancy determine whether an interacting regulatory element is competent to activate or repress transcription, whereas spatial genome organization constrains which regulatory elements can efficiently communicate. Consequently, concordant changes in accessibility and gene expression do not by themselves demonstrate altered 3D architecture.

Epigenetic regulation primarily encompasses DNA methylation, histone acetylation and methylation, alterations in chromatin accessibility, and non-coding RNA-mediated regulation. Among these marks, H3K27ac and H3K4me1 are generally associated with active enhancers, whereas H3K27me3 and H3K9me3 are linked to transcriptional repression and heterochromatin states. DNA methylation can affect the stability of CTCF binding sites and the function of boundary elements, thereby influencing the formation and maintenance of chromatin loops (Wang et al., 2019b). Thus, epigenetic modifications provide the chemical basis by which disease-related genes are switched on or off. Together with 3D chromatin architecture, they form a hierarchical regulatory network.

*In vitro*, inhibition of EZH1/2 with valemetostat, without inducing cell death, markedly altered multiple levels of genome organization in small cell lung cancer, including chromatin compartments, topologically associating domains, and chromatin loops. This treatment also promoted enhancer–promoter looping and reactivated the expression of major histocompatibility complex class I genes (Parveen et al., 2026). Loss of the H3K9 methyltransferase activity of SETDB1 reshaped the epigenetic landscape of both active and inactive chromatin, disrupted higher-order genome organization, activated epithelial transcriptional programs, remodeled the cytoskeleton, reduced proliferative capacity, and constrained cell migration, ultimately reversing the oncogenic potential of lung adenocarcinoma cells (Zakharova et al., 2022). Following acute treatment of lung cancer cells with the histone deacetylase inhibitor suberoylanilide hydroxamic acid (SAHA), gene expression, chromatin accessibility, and 3D chromatin architecture was altered to varying degrees. Notably, although SAHA increased global acetylation levels across the genome, it did not lead to a generalized increase in chromatin accessibility or global opening of higher-order chromatin structure; instead, regulation of local chromatin features supported bidirectional changes in gene expression (Venu et al., 2025). Deletion of Mpr1/Pad1 N-terminal domain-containing protein (MPND) increased H2B K120 ubiquitination and enhanced chromatin accessibility at SMAD3-binding sites, thereby promoting transcriptional activation, tumor growth, and metastasis (Yu et al., 2026).

Multi-omic single-cell analyses of lung tissues from patients with pulmonary fibrosis revealed significant enrichment of TWIST1 and other E-box transcription factor motifs within accessible chromatin regions of idiopathic pulmonary fibrosis (IPF) myofibroblasts (Valenzi et al., 2023). In addition, studies have shown that DNA methylation can regulate local chromatin loop formation by modulating CTCF binding, thereby altering the spatial positioning and transcriptional status of genes. In the Hoxc gene cluster, hypomethylation at specific loci enhanced CTCF occupancy, promoted repositioning of posterior genes toward a more transcriptionally active chromatin environment, and increased their expression, highlighting the coordinated role of epigenetic modifications and 3D chromatin architecture in spatiotemporally specific gene regulation (Min et al., 2016) (Table 1).

**TABLE 1:** Transcription factors and architectural regulators implicated in 3D genome-associated regulation in lung disease.

Factor/regulator	Principal regulatory function	Lung-disease context	Cell/tissue context	3D architectural feature	Reported observation/consequence	Evidence types	References
CTCF	Boundary formation; TAD insulation	Lung development; lung cancer	Embryonic lung; general cell models	TAD boundaries; chromatin loops	Maintains chromatin insulation; supports progenitor maintenance and branching morphogenesis	Direct architectural evidence; developmental functional evidence	(Nora et al., 2017; Sun et al., 2025)
Cohesin/STAG2	Loop extrusion; chromatin topology	Lung cancer	Lung adenocarcinoma cells	Chromatin loops; promoter-enhancer contacts	Supports GR-associated chromatin organization and transcription	Chromatin-interaction and functional evidence	(Rao et al., 2017; Davidson et al., 2019; Mayayo-Peralta et al., 2023)
SMAD3	TGF-β-responsive enhancer regulation	COPD	Bronchial epithelial cells	Enhancer–promoter interaction; local domain organization	Regulates the distal HHIP enhancer and TGF-β-responsive transcription	Chromatin-conformation, enhancer-perturbation, and expression evidence	(Guo et al., 2024)
MYC	Oncogenic transcriptional regulation	Lung cancer	NSCLC/cancer cell models	Enhancer–promoter looping	MYC-related accessibility and distal enhancer-MYC communication promote oncogenic transcription	Accessibility and locus-specific looping evidence	(Wang et al., 2021; Yang et al., 2026)
SMAD4	TGF-β signaling; tumor suppression	Lung squamous cell carcinoma	LUSC cells/models	Loop-anchor; enhancer–promoter interactions	SMAD4 loss enhances EP300-associated interactions and SOX2 activation	Chromatin-interaction and functional evidence	(Tang et al., 2026a)
AP-1/JUN	Stress/inflammatory enhancer regulation	Fibrotic lung disease; lung cancer	Profibrotic fibroblasts; lung cancer cells	Not directly measured	Increased AP-1/JUN-associated enhancer activity and accessibility	Accessibility and functional enhancer evidence	(Wang et al., 2021; Valenzi et al., 2026)
STAT3/NF-κB	Inflammatory/survival regulation	Lung cancer	NSCLC cells	Not directly measured	Motif enrichment during treatment-associated dormancy/reactivation	Supporting epigenomic evidence	(Wang et al., 2021)
RUNX/EGR	Cell-state-specific enhancer regulation	Fibrotic lung disease	Profibrotic fibroblasts	Not directly measured	Increased TF binding and enhancer activity in profibrotic states	Single-nucleus epigenomic evidence	(Valenzi et al., 2026)
GR/PAXIP1	Hormone-responsive chromatin regulation	Lung cancer	Lung adenocarcinoma cells	Promoter-enhancer contacts	PAXIP1/STAG2-dependent interactions support GR-responsive transcription	Chromatin-interaction and functional evidence	(Mayayo-Peralta et al., 2023)

A central unresolved issue is whether the architectural change is a driver, a permissive intermediate, or a consequence of the disease-associated transcriptional state. Cross-sectional correlations among compartment switching, chromatin accessibility, and gene expression cannot distinguish these models. Stronger causal inference is provided when deletion or repression of a specific enhancer alters both topology and transcription, when boundary deletion generates a predictable regulatory phenotype, or when acute depletion and recovery of an architectural protein produce reversible contact changes. Accordingly, future mechanistic studies of lung disease should combine time-resolved 3D mapping with locus-specific perturbation and simultaneous measurement of transcriptional output. Such an approach will allow pathological regulatory networks to be classified according to causality rather than contact correlation alone.

## 5 Current challenges and future perspectives

### 5.1 From static contact maps to temporally and spatially resolved 3D genome measurements

A major limitation of current lung-disease studies is that most chromosome-conformation assays require fixation and provide population-averaged contact frequencies. Conventional Hi-C therefore generates a highly informative but fundamentally ensemble and usually static representation of genome organization. Although comparisons across experimental conditions can identify state-dependent reorganization, they do not directly determine how individual chromatin loci move, how long enhancer–promoter encounters persist, or how rapidly an altered structure is established or reversed. Addressing these questions requires integration of higher-resolution conformation capture, single-cell analysis, spatial imaging, and live-cell tracking (Dekker et al., 2017; Dekker et al., 2023).

Micro-C provides an important extension of chromosome-conformation capture by using micrococcal nuclease fragmentation to generate chromatin contact maps at substantially higher resolution than conventional restriction-enzyme-based Hi-C. In mammalian cells, Micro-C can resolve nucleosome-scale chromatin organization and detect many loops and fine-scale domain features that are poorly resolved by conventional Hi-C (Krietenstein et al., 2020). In lung disease, application of Micro-C to purified disease-relevant cell populations could therefore reveal local enhancer–promoter and boundary alterations that are obscured in lower-resolution bulk maps. Nevertheless, Micro-C remains predominantly a fixed-cell snapshot unless incorporated into a temporal perturbation design.

Single-cell chromatin-conformation methods address a different limitation. Single-cell Hi-C has demonstrated substantial cell-to-cell variability in chromosome structure, while Dip-C enables high-resolution reconstruction of individual diploid genomes and can distinguish homologous chromosomes (Nagano et al., 2013; Tan et al., 2018). These approaches are particularly relevant to heterogeneous lung tissues, where epithelial, stromal, endothelial, and immune cell populations coexist and undergo disease-dependent changes in abundance and state. However, sparse contact coverage and the computational complexity of reconstructing single-cell structures remain important challenges.

Optical methods provide complementary information that cannot be obtained directly from sequencing-based contact frequencies. Multiplexed DNA FISH and optical reconstruction of chromatin architecture (ORCA) can trace multiple genomic positions in individual fixed cells or tissue sections and directly quantify spatial distances and structural heterogeneity (Mateo et al., 2019). ORCA can achieve kilobase-scale genomic tracing with nanoscale spatial localization and can be combined with RNA measurements, thereby linking chromatin structure to transcriptional state at the single-cell level. Importantly, however, ORCA generally requires fixation and should not be described as a real-time live-cell imaging method. Its major strength is high-dimensional spatial reconstruction across many individual cells rather than continuous temporal tracking.

True temporal information can be obtained with CRISPR-based live-cell chromatin imaging. Fluorescently tagged nuclease-deficient Cas9 targeted by guide RNAs enables visualization and tracking of selected endogenous genomic loci in living cells (Chen et al., 2013). Signal-amplification systems such as CRISPR-Sirius improve the brightness and stability of fluorescent labeling and permit multicolor tracking of selected loci (Ma et al., 2018). These approaches can quantify locus trajectories, chromatin mobility, inter-locus distances, and changes across the cell cycle or after stimulation, although multiplexing, signal-to-background ratio, and potential perturbation by the imaging system remain technical considerations.

More recently, Oligo-LiveFISH has extended live-cell imaging to non-repetitive genomic loci using a reagent-based dCas9/guide-RNA strategy. The method was reported to achieve high spatial and temporal resolution and to track dynamic communication between genomic loci in both established and primary cell types (Zhu et al., 2025). Such approaches provide an opportunity to test whether disease-associated enhancer–promoter proximity represents a stable structural state, a transient encounter, or a change in chromatin mobility-questions that cannot be answered from population Hi-C maps alone.

Finally, spatial multi-omics can preserve tissue context while linking epigenetic and transcriptional states. Spatial ATAC-RNA-seq and spatial CUT&Tag-RNA-seq enable joint analysis of chromatin accessibility or histone modifications together with gene expression in the same tissue section (Zhang D. et al., 2023). These methods are particularly attractive for structurally heterogeneous organs such as the lung, because regulatory states can be mapped to epithelial niches, fibrotic lesions, tumor regions, or immune microenvironments. However, they do not directly measure physical chromatin contacts. Their greatest value for 3D genome research is therefore to define the spatial and cellular context in which targeted conformation or imaging assays should be performed.

No single technology can simultaneously provide genome-wide coverage, high spatial resolution, high temporal resolution, cell-type specificity, and direct functional information. A central methodological priority is therefore to combine complementary approaches: high-resolution contact mapping to identify candidate structures, single-cell/spatial multi-omics to define disease-relevant cellular states, live imaging to resolve kinetics, and perturbation experiments to establish causality.

Conventional genome-wide association studies (GWAS) and expression quantitative trait locus (eQTL) analyses identify statistical associations between genetic variants, disease phenotypes, and gene expression but generally do not specify the physical regulatory contacts through which non-coding variants act. Three-dimensional genome approaches can provide this missing spatial constraint for variant-to-gene assignment. H3K27ac HiChIP-based enhancer connectome mapping in primary human cells demonstrated that many disease-associated non-coding elements contact distal promoters, frequently bypassing the nearest gene in the linear genome (Mumbach et al., 2017). A pulmonary-disease example is provided by the 17q21 asthma-risk locus, where allele-dependent changes in CTCF occupancy were accompanied by altered 4C-seq contacts between distal cis-regulatory elements and the ORMDL3 promoter (Schmiedel et al., 2016). Chandra et al. subsequently integrated H3K27ac HiChIP with eQTL mapping to define promoter-interacting eQTLs (pieQTLs), thereby identifying cell-type-specific and ultra-long-range regulatory variants that can be missed or ambiguously assigned by conventional distance-based approaches (Chandra et al., 2021). In lung disease, integration of Hi-C, ChIP-seq, eQTL analysis, and CRISPRi has similarly linked non-coding disease-associated loci to specific regulatory targets, including TGFB1 and MUC5AC/MUC5B (Stuart et al., 2020). Collectively, these approaches illustrate how three-dimensional chromatin information can refine variant-to-gene assignment and prioritize candidate mechanisms for functional validation. However, translation into precision medicine will require validation in disease-relevant primary lung cell populations and patient samples, because chromatin interactions are strongly cell-type- and state-dependent and physical proximity alone does not establish causality.

### 5.2 Future perspectives: from structural maps to causal and translational 3D genome biology

The next phase of 3D genome research in lung disease should move beyond cataloguing structural differences and address several fundamental biological questions.

First, the causal direction between chromatin architecture and transcription remains unresolved at many disease-associated loci. Increased enhancer–promoter contact can precede transcriptional activation, facilitate it, stabilize it, or arise as a consequence of transcription-factor recruitment and chromatin-state change. Resolving these alternatives will require experiments in which architecture and transcription are measured simultaneously following precisely timed perturbations.

Second, the temporal stability and reversibility of disease-associated 3D states remain largely unknown. Chronic lung diseases are characterized by repeated or persistent exposures to cigarette smoke, inflammatory cytokines, oxidative stress, hypoxia, tissue injury, and therapeutic interventions. It will therefore be important to determine whether pathological chromatin configurations rapidly revert after removal of a stimulus or whether some structures persist as a form of regulatory “memory” that predisposes cells to recurrent inflammation, fibrosis, or treatment resistance. Time-resolved mapping and live-cell tracking will be essential for distinguishing transient responses from stable architectural reprogramming (Dekker et al., 2023).

Third, 3D genome remodeling must be resolved at the level of specific cell types and disease stages. Bulk lung tissue is especially problematic because apparent architectural differences can result from changes in cellular composition. An increase in a contact detected in fibrotic tissue, for example, may reflect increased abundance of a fibroblast population rather than *de novo* formation of the interaction within each fibroblast. Integration of single-cell chromatin profiling, spatial multi-omics, and purified-cell conformation assays will therefore be necessary to distinguish population shifts from cell-intrinsic remodeling.

Fourth, a major opportunity lies in connecting genotype to spatial regulatory phenotype. Most common disease-associated variants reside outside coding regions, and structural variants are particularly capable of altering regulatory topology. Allele-resolved 3D genome analysis could help determine whether a risk allele changes enhancer activity, boundary strength, nuclear positioning, or promoter contact in a cell type-specific manner. Integrating genetic variation with epigenomic state, 3D contacts, and transcription may therefore help explain why individuals carrying similar risk variants develop different disease trajectories.

Fifth, translation will require identifying actionable rather than merely abnormal chromatin interactions. Because CTCF and cohesin perform widespread and essential functions, global pharmacological disruption of architectural proteins would be expected to have broad genomic consequences and is unlikely to provide an optimal disease-selective strategy. More plausible approaches include locus-specific CRISPRi or epigenome editing of pathological enhancers, selective inhibition of transcription factors or cofactors required to maintain disease-specific regulatory interactions, and pharmacological modulation of epigenetic enzymes that establish permissive enhancer states. Disease-associated 3D architecture may also eventually contribute to molecular stratification or prediction of therapeutic response, particularly when integrated with genetic and single-cell data. However, such applications will require prospective validation and demonstration that the candidate architectural feature is sufficiently stable, disease-relevant, and measurable in clinically accessible material.

Ultimately, the field will need predictive models capable of integrating genetic variation, chromatin state, transcription-factor occupancy, nuclear position, 3D contacts, and gene expression across cell types and time. The most informative models will be those that predict the transcriptional consequence of a defined architectural perturbation and can subsequently be experimentally tested. Such a transition from descriptive maps to predictive and perturbation-validated models is likely to determine whether 3D genome biology becomes mechanistically and clinically useful in lung disease.

## 6 Conclusion

3D chromatin architecture provides an organizational framework through which genetic variation, epigenetic state, transcription-factor activity, and distal regulatory elements are converted into cell type-specific transcriptional outputs. Across lung diseases, current evidence indicates that pathological gene regulation can be associated with multiple levels of spatial genome reorganization, including compartment switching, altered domain insulation, chromatin-loop rewiring, and changes in enhancer–promoter communication. These structural changes are not mechanistically equivalent. Chromatin-state-dependent compartmental segregation and CTCF/cohesin-mediated loop extrusion represent partially separable organizing principles, and their relative contributions differ among disease contexts.

A cross-disease comparison also reveals both convergence and divergence. COPD illustrates how non-coding susceptibility loci can alter distal regulatory communication; pulmonary fibrosis highlights the interaction between chromatin state, cellular heterogeneity, and profibrotic transcriptional programs; lung cancer currently provides the richest evidence for oncogenic enhancer rewiring and altered regulatory topology; and developmental studies demonstrate that selected chromatin boundaries can be causally required for normal lung morphogenesis. These observations support a general model in which disease-associated transcription factors operate within a remodelable spatial framework, while architectural proteins constrain the range and specificity of regulatory interactions.

At the same time, the field should distinguish evidence for altered chromatin states from evidence for true temporal dynamics. Most current lung-disease studies remain based on cross-sectional comparisons and therefore demonstrate state-dependent reorganization rather than chromatin kinetics. The major challenge is now to determine which architectural abnormalities are causal, which are reversible, which are specific to individual cell populations, and which are consequences of altered transcription or tissue composition.

Future progress will therefore depend on integrating cell type-resolved 3D genome profiling, high-resolution conformation capture, spatial multi-omics, live-cell chromatin imaging, and targeted perturbation. Moving from static maps to temporally resolved and experimentally testable models should clarify how pathological regulatory circuits are established and maintained. If disease-specific chromatin interactions can be shown to be causal, measurable, and selectively targetable, 3D genome biology may ultimately contribute to mechanistically informed patient stratification and more precise therapeutic intervention in lung disease.
